# Solution NMR structures of proteins VPA0419 from *Vibrio parahaemolyticus* and yiiS from *Shigella flexneri* provide structural coverage for protein domain family PFAM 04175

**DOI:** 10.1002/prot.22630

**Published:** 2009-10-16

**Authors:** Kiran Kumar Singarapu, Jeffrey L Mills, Rong Xiao, Thomas Acton, Marco Punta, Markus Fischer, Barry Honig, Burkhard Rost, Gaetano T Montelione, Thomas Szyperski

**Affiliations:** 1 Department of Chemistry, State University of New York at BuffaloBuffalo, New York 14260; 2 Northeast Structural Genomics Consortium; 3 Center of Advanced Biotechnology and Medicine, Rutgers UniversityPiscataway, New Jersey 08854; 4 Department of Molecular Biology and Biochemistry, Rutgers UniversityPiscataway, New Jersey 08854; 5 Department of Biochemistry and Molecular Biophysics, Columbia UniversityNew York, New York 10032; 6 Computational Biology and Bioinformatics (C2B2), Columbia UniversityNew York, New York 10032; 7 Howard Hughes Medical Institute, Center for Computational Biology and Bioinformatics, Columbia UniversityNew York, New York 10032

**Keywords:** VPA0419, yiiS, PFAM 04175, structural genomics, GFT NMR

## INTRODUCTION

Protein with 83 residues, VPA0419 (residues 17–99, numbered 1–83) (gi|81726230, SwissProt/TrEMBL ID Q87J34_VIBPA, accession number Q87J34)^1^ from *Vibrio parahaemolyticus* and 99-residue protein yiiS (gi|81722782, SwissProt/TrEMBL ID Q83IT9_SHIFL, accession number Q83IT9)^2^, ^3^ from *Shigella flexneri* were selected as targets for the Protein Structure Initiative-2 and assigned to the Northeast Structural Genomics Consortium (NESG) for structure determination (NESG target ID VpR68 for VPA0419 and SfR90 for yiiS). VPA0419 and yiiS share 36% sequence identity, but show no significant sequence identity with any protein with known three-dimensional structure in the Protein Data Bank (PDB^4^). The two proteins belong to Pfam^5^ domain family PF04175 which currently contains 123 members with unknown three-dimensional structures and functional annotation, all of which appear to be found in *gamma proteobacteria* (for a sequence alignment, see Fig. S1 in Supporting Information). The NMR structures of VPA0419 and yiiS were solved using a protocol for high-throughput protein structure determination^6^ and represent the first ones for protein family PF04175. As these structures are the first for PF04175, “high leverage”^7^ of the experimental structures can be expected for calculating homology models.^8^, ^9^

## METHODS

Proteins VPA0419 and yiiS were cloned, expressed, and purified following standard protocols developed by the NESG for production of *U*-^13^C,^15^N-labeled protein samples.^10^ Briefly, the truncated VPA0419 gene encoding residues 17–99 from *Vibrio parahaemolyticus* and the full-length yiiS gene from *Shigella flexneri* were cloned into a pET21 (Novagen) derivative, yielding the plasmids VpR68-17-99-21.2 and SfR90-21.8, respectively. The resulting constructs contain eight nonnative residues at the C-terminus (LEHHHHHH) that facilitate protein purification. The construct of VPA0419 was designed using consensus disorder prediction methods (Y. Huang and G.T. Montelione, personal communcation); several constructs with different truncations of the N-terminal 14–22 residues were made, and the construct providing the best expression, solubility, and NMR spectral quality, residues 17–99, was selected for structure determination. *Escherichia coli* BL21 (DE3) pMGK cells, a codon enhanced strain, were transformed with VpR68-17-99-21.2 for protein VPA0419 and SfR90-21.8 for protein yiiS, and cultured in MJ9 minimal medium containing (^15^NH_4_)_2_SO_4_ and *U*-^13^C-glucose as sole nitrogen and carbon sources to produce *U*-^13^C,^15^N-labeled proteins. VPA0419 and yiiS were purified using an AKTAxpress (GE Healthcare) based two-step protocol consisting of IMAC (HisTrap HP) and gel filtration (HiLoad 26/60 Superdex 75) chromatography. The final yields of purified *U*-^13^C,^15^N VPA0419 (>98% homogenous by SDS-PAGE; 10.8 kDa by MALDI-TOF mass spectrometry) and *U*-^13^C,^15^N yiiS (>98% homogenous by SDS-PAGE; 12.4 kDa by MALDI-TOF mass spectrometry) were ∼43 and ∼34 mg/L, respectively. In addition, *U*-^15^N, 5% biosynthetically directed fractionally ^13^C-labeled samples were generated to stereo-specifically assign Val and Leu methyl groups.^11^ All NMR samples were prepared at ∼1 m*M* protein concentration in 95% H_2_O/5% ^2^H_2_O solutions containing 20 m*M* NH_4_OAc (VPA0419) or 20 m*M* MES (yiiS) along with 100 m*M* NaCl, 5 m*M* CaCl_2_, 10 m*M* DTT, and 0.02% NaN_3_ at pH 5.5 (VPA0419) or pH 6.5 (yiiS). Isotropic overall rotational correlation times of ∼4.5 ns for VPA0419 and ∼5.0 ns for yiiS were inferred from ^15^N spin relaxation times and indicate that both proteins are monomeric in solution. This conclusion was further confirmed by analytic gel filtration (Agilent Technologies) followed by a combination of static light scattering and refractive index (Wyatt Technology).^10^

NMR spectra were recorded at 25°C on Varian INOVA 600 (for VPA0419) and INOVA 750 (for yiiS) spectrometers equipped with cryogenic probes. Four through-bond correlated G-matrix Fourier transform^12^ (GFT) NMR experiments,^12^–^14^ complemented by 3D HNNCO as described,^15^ were collected for backbone and side chain resonance assignments (total measurement time: ∼100 h for each protein). For both proteins, simultaneous 3D ^15^N/^13^C^aliphatic^/^13^C^aromatic^-resolved [^1^H,^1^H]-NOESY^14^ (mixing time: 70 ms; measurement time: 24 h for each protein) was acquired on an a Varian INOVA 750 spectrometer to derive ^1^H–^1^H distance constraints. 2D constant-time [^13^C,^1^H]-HSQC spectra were recorded as was described^15^ for the 5% fractionally ^13^C-labeled samples in order to obtain stereo-specific assignments for isopropyl groups of Val and Leu. Spectra were processed and analyzed with the programs NMRPIPE^16^ and XEASY,^17^ respectively.

Sequence specific backbone (^1^H^N^, ^15^N, ^1^H^α^, ^13^C^α^) and ^1^H^β^/^13^C^β^ resonance assignments were obtained by using (4,3)D HNNC^αβ^C^α^/C^αβ^C^α^(CO)NHN and H^αβ^C^αβ^(CO)NHN along with the program AUTOASSIGN,^18^ and polypeptide backbone ^13^C′ resonances were assigned using 3D HNNCO. More peripheral side chain chemical shifts were assigned with aliphatic (4,3)D HCCH and 3D ^15^N/^13^C^aliphatic^/^13^C^aromatic^-resolved [^1^H,^1^H]-NOESY. Overall, assignments were obtained for 100%/99% of backbone and ^1^H^β^/^13^C^β^ resonances of VPA0419/yiiS, and for 99% of the side chain resonances of both proteins which are assignable with the NMR experiments listed earlier (excluding the N-terminal NH_3_^+^, Pro ^15^N, ^13^C′ preceding prolyl residues, Lys NH_3_^+^, Arg NH_2_, OH, side chain ^13^C′, and aromatic ^13^C^γ^). Furthermore, 64%/100% of Val and Leu isopropyl moieties and 49%/28% of β-methylene groups with non-degenerate proton chemical shifts were stereo-specifically assigned for VPA0419/yiiS (Table I). Chemical shifts were deposited in the BioMagResBank^22^ (accession code 15608 for VPA0419 and 15762 for yiiS). ^1^H–^1^H upper distance limit constraints for structure calculations were obtained from NOESY (Table I). In addition, backbone dihedral angle constraints were derived from chemical shifts using the program TALOS^23^ for residues located in well-defined secondary structure elements (Table I). The programs CYANA^24^, ^25^ and AUTOSTRUCTURE^26^ were used in parallel to assign long-range NOEs.^6^ The final structure calculations were performed using CYANA followed by explicit water bath refinement using the program CNS.^27^

**Table I:** Structure Statistics for NMR Structures of Proteins VPA0419 and yiiS

	VPA0419	yiiS
Completeness of stereospecific assignments^a^ (%)		
^β^CH_2_	49 (27/55)	28 (11/39)
Val and Leu methyl groups	64 (9/14)	100 (9/9)
Conformationally restricting distance constraints		
Intraresidue [*i* = *j*]	439	346
Sequential [|*i – j*| = 1]	493	588
Medium range [1 < |*i – j*| < 5]	323	443
Long range [|*i – j*| > 5]	554	621
Total	1809	1998
Dihedral angle constraints		
φ	45	47
ψ	45	47
Average number of constraints per residue	21.0	20.2
Average number of long-range distance constraints per residue	6.4	6.3
CYANA target function (Å^2^)	0.95 ± 0.20	0.90 ± 0.13
Average number of distance constraints violations per CYANA conformer		
0.2–0.5 Å	0	0
>0.5 Å	0	0
Average number of dihedral-angle constraint violations per CYANA conformer		
>5°	0	0
Average rmsd to the mean CNS coordinates (Å)		
Regular secondary structure elements,^b^ backbone heavy atoms	0.48 ± 0.10	0.57 ± 0.10
Regular secondary structure elements,^b^ all heavy atoms	0.92 ± 0.10	1.00 ± 0.06
Ordered residues,^c^ backbone heavy atoms	1.21 ± 0.24	0.73 ± 0.11
Ordered residues,^c^ all heavy atoms	1.48 ± 0.18	1.12 ± 0.09
Heavy atoms of molecular core including best-defined side chains^d^	0.73 ± 0.13	0.86 ± 0.09
PROCHECK^19^ G-factors raw score (φ and ψ / all dihedral angles)^c^	−0.09/−0.13	−0.08/−0.11
PROCHECK^19^ G-factors Z-score (φ and ψ / all dihedral angles)^c^	−0.04/−0.77	0.00/−0.65
MOLPROBITY^20^ clash score (raw / *Z*-score)^c^	19.82/−1.90	19.26/−1.78
AutoQF R/P/DP scores^21^ (%)	95/97/78	93/96/71
Ramachandran plot summary^c^ (%)		
Most favored regions	95.1	94.7
Additionally allowed regions	4.9	5.2
Generously allowed regions	0.0	0.1
Disallowed regions	0.0	0.1

a Relative to pairs with nondegenerate chemical shifts for residues 1–83 (VPA0419) and 29–100 (yiiS).

b Residues 14–19, 24–40, 46–49, 58–64, 68–81 for VPA0419; 29–36, 39–56, 64–72, 75–83, 87–100 for yiiS.

c Residues 1-83 for VPA0419; 29-100 for yiiS.

d Backbone and side-chain heavy atoms of residues 14,15,17–19, 26, 27, 29, 31, 32, 35, 38, 40, 41, 45, 48, 50, 59–61, 69, 72, 73, 76 for VPA0419; 29, 36, 39, 42–46, 52, 53, 56, 69–71, 75–84, 89–93 for yiiS.

Computational structure analyses are provided by the NESG function annotation database (http://luna.bioc.columbia.edu/honiglab/nesg). This resource provides information on structural neighbors identified by the structure alignment methods SKAN^28^, ^29^ and DALI,^30^ sequence neighbors extracted from UniProt^31^ using PSI-BLAST,^32^ solvent accessible cavities identified by SCREEN,^33^ electrostatic surface potentials estimated by DELPHI,^34^ protein signatures recognized by INTERPROSCAN,^35^ and amino acid conservation profiles estimated by CONSURF.^36^ For structure visualization, the ASTEXVIEWER™ 2.0^37^ is implemented. Further details on the applied methods are provided at http://luna.bioc.columbia.edu/honiglab/nesg/documentation/.

## RESULTS AND DISCUSSION

**Figure 1:**
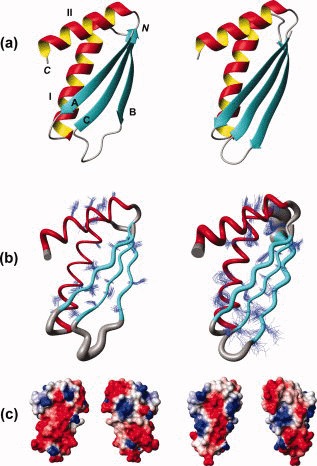
NMR structures of proteins VPA0419 (residues 13–82; on the left) and yiiS (residues 28–101; on the right). (**a**) Ribbon drawing of the conformer with the lowest CYANA target function. The α-helices I and II are shown in red and yellow, β-strands A, B, and C are shown in cyan, and other polypeptide segments are shown in grey. The N- and C-termini are labeled with “N” and “C,” respectively. (**b**) “Sausage” representation of backbone and best-defined side chains (Table I). A spline curve was drawn through the mean positions of C^α^ atoms of the residues of the regular secondary structure elements with the thickness proportional to the mean global displacement of C^α^ atoms in the 20 conformers representing the NMR structures (Table I) superimposed for minimal rmsd. The coloring is as in (a). (**c**) Electrostatic surface potential. The left of the two representations for each of the two proteins is in the same orientation as (a) and (b). The corresponding presentations on the right were obtained after a 180° rotation about the vertical axis. The figures were generated with the program MOLMOL.^38^

High-quality three-dimensional NMR structures (Table I) were obtained for proteins VPA0419 and yiiS, and the coordinates were deposited in the PDB^4^ (Fig. 1; accession code 2jz5 for VPA0419 and 2k3i for yiiS). As was expected for two proteins with 36% sequence identity, both proteins exhibit quite similar three-dimensional structures: the root mean square deviation (rmsd) calculated between the mean coordinates of the backbone heavy atoms N, C^α^, and C′ of regular secondary structure elements is only 0.88 Å. The two proteins exhibit a mixed α/β fold containing a three-stranded antiparallel β-sheet with topology A(↑)C(↓)B(↑) and comprising residues 14–20/29–36, 45–50/63–71, and 58–65/76–83. Two α-helices I and II, comprise, respectively, residues 24–41/39–59 and 68–82/87–101, and are attached on one side of the β-sheet. α-Helix I is inserted between β-strands A and B, while α-Helix II is C-terminal.

As mentioned in the introduction, both VPA0419 and yiiS belong to protein domain family PF04175,^5^ a “domain of unknown function” family (DUF406) whose members appear to be found only in *gamma proteobacteria*. Although PSI-BLAST^32^ searches using VPA0419 and yiiS as queries against UniRef100^39^ returned a few closely related annotated sequences [e.g. a sequence annotated as putative C-4 dicarboxylate transport protein (fragment) or one annotated as DNA mismatch repair protein sharing 98% and 36% sequence identity, respectively, with yiiS over the entire protein length], it was unclear why these sequences carried such annotations in UniRef and we could not validate them. To the best of our knowledge, the only tentative functional annotation published in literature for any member of family PF04175 refers to a *Haemophilus influenzae* protein (Swiss-Prot ID P44027; 25% sequence identity with protein yiiS over 84 residues). This protein has been suggested to play an auxiliary role in penetration of the bacterial cells in between human epithelial cells.^40^ However, the specific mechanism by which this protein is involved is not known, so that a biochemical functional annotation of members of PF04175 can likewise not be derived.

Our search for proteins that are structurally similar to VPA0419 and yiiS (see Supporting Information for additional details) reveals that their three-dimensional architecture is rather common among proteins with known structures deposited in the PDB.^4^ For example, the structure alignment programs SKAN^28^, ^29^ and DALI^30^ return numerous ‘hits’ when using VPA0419 and yiiS as queries, including several RNA binding domains such as the spliceosomal U2B“ protein^41^ and the S10 component of the 30S ribosomal subunit.^42^ However, the RNA binding region in these domains is generally located on the solvent exposed side of the β-sheet, an area that in VPA0419 and yiiS exhibits only slightly positive to neutral surface electrostatic potential (GRASP2^28^). Furthermore, potential RNA binding residues appear in general not to be conserved in VPA0419 and yiiS. Although this does not necessarily rule out the possibility that the two proteins bind to RNA, a functional annotation for VPA0419 and yiiS can thus not be inferred from these structurally similar proteins.

The only structural match for VPA0419 and yiiS for which we could derive a tentative suggestion for a functional annotation turned out to be the single domain enzymes Pterin-4a-Carbinolamine Dehydratases (PCDs).^43^ The structure of the *Thermus thermophilus* protein DCoH^44^ can be superposed onto yiiS and VPA0419 with, respectively, r.m.s.d. values for superposition of the C^α^ atoms of 2.8 Å (70 aligned residues) and 3.1 Å (66 aligned residues) when using the program SKAN.^28^, ^29^ The sequence identity inferred from a structure-based sequence alignment, on the other hand, is very low: 11% and 12% for, respectively, yiiS and VPA0419. Very similar results are obtained for the mouse and rat homologs (PDB IDs 1ru0^45^ and 1dcp^46^). Hence, the hypothesis presented in the following could not have been drawn based on sequence similarity alone and depended on the knowledge of the three-dimensional structures.

Although residues of the PCDs involved in binding of the metabolite pterin-4a-carbinolamine are not conserved in VPA0419 and yiiS, they do correspond to a surface region structurally aligned with residues that are highly conserved in PF04175 (in particular also the most conserved residue Glu 89; see Supporting Information with additional information and Fig. S2). This suggests that in both VPA0419 and yiiS (and therefore all members of PF04175), this region (i.e. the one including Glu 89) is involved in binding of a ligand, thereby possibly constituting a catalytic site of a yet uncharacterized enzyme specific to *gamma proteobacteria*. Future experiments designed to screen for ligands that bind to proteins VPA0419 and/or yiiS can be envisaged to test this hypothesis.
