# Supplementary material for: Solution NMR structures of proteins VPA0419 from Vibrio parahaemolyticus and yiiS from Shigella flexneri provide structural coverage for protein domain family PFAM 04175
Source: Proteins. 2009 Oct 16;78(3):779–84. doi: 10.1002/prot.22630 (PMC2860719; doi:10.1002/prot.22630)
Supplement: Supplementary file 1 [file prot0078-0779-SD1.pdf]

Supporting Information for

# Solution NMR structures of proteins VPA0419 from *Vibrio parahaemolyticus* and yiiS from *Shigella flexneri* provide structural coverage for protein domain family PFAM 04175

**Kiran Kumar Singarapu,[1,6†] Jeffrey L. Mills,[1,6†] Rong Xiao,[2,6] Thomas Acton,[2,6] Marco Punta[3,4,6], Markus Fischer,[3,6] Barry Honig,[3,5,6] Burkhard Rost,[3,4,6] Gaetano T. Montelione,[2,6] and Thomas Szyperski[1,6*]**

```
Q87J34_VIBPA/18-95    GEIGFIIKEGDEVADVTIFA-ETKDALESELAKYIELAKSVCA---- GVEYNVSELTEESKELTARFKFEVSAEKLIFELKTR
Q83IT9_SHIFL/15-96    IDIGTVIDNDNCTSKFSRFF-ATREEAESFMTKLKELAAAASSADEGASVAYKIKDL-EGQVELDAAFTFSCQAEMIIFELSLR
A4N8Z5_HAEIN/16-95    VDVGTIIDGSDCSVEVHQFY-STEADANAALERLTKKARDTES--DPCEIKSEIVAV-ENGVQLNASFTFSCQAEAMIFELANR
A4NJ91_HAEIN/2-53     -----------------------------ALERLTKKARDTES--DPCEIKSEIVAV-ENGVQLNASFTFSCQAEAMIFELANR
Q4QNE2_HAEI8/16-95    VDVGTIIDGSDCSVEVHQVY-STEADANAALERLTKKARDIES--DPCEIKSEIVAV-ENGVQLNASFTFSCQAEAMIFELANR
A4MY27_HAEIN/16-95    VDVGTIIDGSDCSVEVHQVY-STEADANAALERLTKKARDIES--DPCEIKSEIVAV-ENGVQLNASFTFSCQAEAMIFELANR
A4NR22_HAEIN/16-95    VDVGTIIDGSDCTVEVHQFY-STEADANAALERLTKKARDIES--DPCEIKSEIVAV-ENGVQLNASFTFSCQAEAMIFELANR
A5UGR6_HAEIG/16-95    VDVGTIIDGSDCSVEVHQFY-STEADANAALERLMKKARNTES--DPCEIKSEIVAV-ENGVQLNASFTFSCQAEAMIFELANR
A5UA88_HAEIE/16-95    VDVGTIIDGSDCSVEVHQFY-STEADANAALERLTKKARNTES--DPCEIKSEIVAV-ENGVQLNASFTFSCQAEAMIFELANR
Y400_HAEIN/16-95      VDVGTIIDGSDCSVEVHQFY-STEADANAVLERLTKKARNTES--DPCEIKSEIVAV-ENGVQLNASFTFSCQAEAMIFELANR
A4NEQ4_HAEIN/16-95    VDVGTIIDGSDCSVEVHQVY-STEADANAALERLTKKARNTES--DPCEIKSEIVAV-ENGVQLNASFTFSCQAEAMIFELANR
A4NVS0_HAEIN/16-95    VDVGTIIDGSDCSVEVHQVY-STEADANAALERLIKKARDTES--DPCEIKSEIVAV-ENGVQLNVSFTFSCQAEAMIFELANR
Q0I2K6_HAES1/19-98    IKGDSMLDNSAREVVFEAEY-DTEKQALNARDYFIQKARDIEN--DPANIESQITDI-SNGFRLKMRIVFGCQAEVVLFQMAIR
A7JX79_PASHA/18-97    LKGDSMLDNADRQIAFEAVY-ESEELAQQAVEFFAQKAKSVET--EPCEIQSEITQI-AEGFLMKMWITFSCQAEVILFQMAVR
A3N259_ACTP2/18-97    LKGDSMLDNSDRQIAFEAVY-DSEEQAQQAVEFFTEKAKSVET--EACVIQHSISPI-EDGFLMQMRIEFSCQAEVVLFQMAVR
A4P1H9_HAEIN/4-34     ----------------------------------------------------EITKV-DDGYLLKADFTFCCQAELVIFQMRIA
A5UEA0_HAEIE/1-78     --MKSLFDNRDCSQVIEYIY-DSEEQAQEALAFFTQKARDVES--EPCEIQSEITKV-DDGYLLKADFTFCCQAELVIFQMRIA
Q4QMT1_HAEI8/17-96    FDMKSLFDNRDCSQVIEYIY-DSEEQAQEALAFFTQKARDVES--EPCEIQSEITKL-DDGYLLKADFTFCCQAELVIFQMRIA
A4P0W2_HAEIN/17-62    FDMKSLFDNRDCSQVIEYIY-DSEEQAQEALAFFTQKARDVES--EPCE-----------------------------------
A4NHJ5_HAEIN/17-96    FDMKSLFDNRDCSQVIEYIY-DSEEQAQEALAFFTQKARDVES--EPCEIQSEITKV-DDGYLLKADFTFCCQAELVIFQMRIA
A4N7B4_HAEIN/17-96    FDMKSLFDNRDCSQVIEYIY-DSEEQAQEALAFFTQKARDVES--EPCEIQSEITKV-DDGYLLKADFTFCCQAELVIFQMRIA
A4NCG9_HAEIN/17-96    FDMKSLFDNRDCSQVIEYIY-DSEEQAQEALAFFTQKARDVES--EPCEIQSEITKV-DDGYLLKADFTFCCQAELVIFQMRIA
A4NT33_HAEIN/17-96    FDMKSLFDNRDCSQVIEYIY-DSEEQAQEALAFFTQKARDVES--EPCEIQSEITKV-DDGYLLKANFTFCCQAELVIFQMRIA
A4N0Q3_HAEIN/17-96    FDMKSLFDNRDCSQVIEYIY-DSEEQAQEALAFFTQKARDVES--EPCEIQSEITKV-DDGYLLKANFTFCCQAELVIFQMRIA
A5UHC7_HAEIG/17-96    FDMKSLFDNRDCSQVIEYIY-DSEEQAQEALAFFIQKARDVES--EPCEIQSEITKV-DDGYLLKADFTFCCQAELVIFQMRIA
Y636_HAEIN/17-96      FDMKSLFDNRDCSQVIEYIY-DSEGQAQEALAFFTQKARDVES--EPCEIQSEITKV-DDGYLLKADFTFCCQAELVIFQMRIA
A4NP03_HAEIN/17-96    FDMKSLFDNRDCSQVIEYVY-DSEEQAQEALAFFTQKARDVES--EPCEIQSEITKV-DDGYLLKADFTFCCQAELVIFQMRIA
A4N692_HAEIN/17-96    FDVKSLFDNRDCSQVIEYIY-DSEEQAQEALAFFTQKARDVES--EPCEIQSEITKV-DDGYLLKANFTFCCQAELVIFQMRIA
A4NT36_HAEIN/4-37     -------------------------------------------------ITTEITKV-DDGYLLKADFTFCCQAELVIFQMRIA
A7JS05_PASHA/15-94    FDVGSILDNSETVATIQRTY-ESENEAKAALEKFTQKARDVES--EPCEISSEITPI-ESGFELVAHFKFSCQAEVVIFQLGTR
A3N2Q6_ACTP2/15-94    FDVGSIIDNSERDAKFEKVY-ETQAEAEQALVKLTQKARDTES--EPCEITSEIKAV-EGGFLLDANFRFSCQAEVVIFQLGTR
Q7VKK6_HAEDU/57-136   FDVGSIIDNSERDAKFEKVY-ATQEQAEAVLAKLVQKARDTET--DSCEISSNILPV-EAGFLLTANFDFSCQAEVVIFQLGTR
Q65Q92_MANSM/16-95    FDVGSILNNDELEAKIEKVF-AGKEEAEQGLAALTAKARDIES--EPCKISSEITPV-DGGYKLTASFEFSCQAEVVIFQLGTR
A6VKP7_ACTSC/15-94    FDVGSILDNSDLKADFDKVY-PTRAEAEAALAKLTQKARDTET--EPCQIQSEINEA-EGGFRLKAGFVFSCQAEAVIFQLGTR
A6D6P5_9VIBR/18-95    GEMGFIIKEGDDVAEVQLFS-DSKAQLEVELQKYVELAQAVCK-----NVEFEVSPLEESSKELTARFKFEVSAEKLIFELKTR
A7N647_VIBHB/18-95    GEMGFIIKEGDDVADVTIFA-ETKAALEAELAKYIEMATAVCA-----GVEYNVSELTEESKELTARFKFEVSAEKLIFELKTR
Q1VF95_VIBAL/18-95    GEIGFIIKEGDEVADVTVFA-QTKTELESELQKYIELAKSVCA-----DVEYNVSEITEESNQATARFKFEVSAEKLIFELKTR
A7K2E8_9VIBR/18-95    GEIGFIIKEGDEVADVTVFA-QTKTELESELQKYIELAKSVCA-----DVEYNVSEITEESNQATARFKFEVSAEKLIFELKTR
Q8D3T0_VIBVU/18-95    GEIGFIIKEGDDVADVTIYA-GSKELLQAEFAKYLELAKAVNE-----NVEYNTTDLTDDSTELTARFKFEVSAEKLIFELKSR
Q7MFA3_VIBVY/18-95    GEIGFIIKEGDDVADVTIYA-GSKELLQAEFAKYLELAKAVNE-----NVEYNTTDLTDDSTELTARFKFEVSAEKLIFELKSR
A5KTU3_9GAMM/18-95    GEIGFIIKEGDDVAEVTVYG-NSKALIEAEFAKYVELAKQVSS-----NVEYEASEMTEESTELHARFKFEVSAEKIIFELKTR
A3XTE3_9VIBR/18-95    GEIGFIIKEGDDVAEVTVYG-NSKALIETEFAKYVELAKQVSS-----NVEFEASELTEESTELNARFKFEVSAEKIIFELKTR
A3ULA3_VIBSP/18-95    GEIGFIIKEGDEVAEVTVYG-NSKALIEAELAKYVELAKQVSS-----NVEFEASEMTEDSTELHARFKFEVSAEKIIFELKTR
A3E9A0_VIBCH/19-96    GEIGFIIREGDDVAEVSLFG-SDKAHLEGKLAEYISLAKQVYA-----NVEYEVAPVADNATELHARFKFEVSAEKLIFELKTR
Q9KL30_VIBCH/19-96    GEIGFIIREGDDVAEVSLFG-SDKAHLEGKLAEYISLAKQVYA-----NVEYEVAPVADNATELHARFKFEVSAEKLIFELKTR
A3GW36_VIBCH/19-96    GEIGFIIREGDDVAEVSLFG-SDKAHLEGKLAEYISLAKQVYA-----NVEYEVAPVADNATELHARFKFEVSAEKLIFELKTR
A1EJI6_VIBCH/19-96    GEIGFIIREGDDVAEVSLFG-SDKAHLEGKLAEYISLAKQVCA-----NVEYEVAPVADNATELHARFKFEVSAEKLIFELKTR
A2P454_VIBCH/19-96    GEIGFIIREGDDVAEVSLFG-SDKAHLEGKLAEYISLAKQVCA-----NVEYEVAPVADNATELHARFKFEVSAEKLIFELKTR
A1F3K2_VIBCH/19-96    GEIGFIIREGDDVAEVSLFG-SDKAHLEGKLAEYISLAKQVCA-----NVEYEVAPVADNATELHARFKFEVSAEKLIFELKTR
A6A054_VIBCH/19-96    GEIGFIIREGDDVAEVSLFG-SDKAHLEGKLAEYISLAKQVCA-----NVEYEVAPVADNATELHARFKFEVSAEKLIFELKTR
A2PRE4_VIBCH/19-96    GEIGFIIREGDDVAEVSLFG-SDKAHLEGKLAEYISLAKQVCA-----NVEYEVAPVADNATELHARFKFEVSAEKLIFELKTR
A6XQL6_VIBCH/19-96    GEIGFIIREGDDVAEVSLFG-SDKAHLEGKLAEYISLAKQVCA-----NVEYEVAPVADNATELHARFKFEVSAEKLIFELKTR
A5F0S1_VIBCH/19-96    GEIGFIIREGDDVAEVSLFG-SDKAHLEGKLAEYISLAKQVCA-----NVEYEVAPVADNATELHARFKFEVSAEKLIFELKTR
A6A9K5_VIBCH/19-96    GEIGFIIREGDDVAEVSLFG-SDKAHLEGKLAEYISLAKQVCA-----NVEYEVAPVADNATELHARFKFEVSAEKLIFELKTR
A3GN70_VIBCH/19-96    GEIGFIIREGDDVAEVSLFG-SDKAHLEGKLAEYISLAKQVCA-----NVEYEVAPVADNATELHARFKFEVSAEKLIFELKTR
Q1ZWZ6_9VIBR/18-95    GELGFIIKENDDTADVQIFA-QGKDALEVELANYLALAKEVNA-----NYKVETTPVDENSTELTARIQFECSAEKLIFELRSR
Q2C8E1_9GAMM/18-95    AEMGFIIKENDDTADVKVFA-NGKDALEIELSNYLALAKEVNA-----NYKVETTPVNADSTELTARIQFECSAEKLIFELRSR
Q1Z722_PHOPR/18-95    AEMGFIIKEGDDVAEVSIFA-QSKVALENEFNNYLALAKEVNA-----NFEYQVSELTEESTELTARIKFECSAEKLIFELKSR
Q6LK04_PHOPR/18-95    AEVGFIIKEGDDVAEVSIFA-QGKVALENEFNNYLALAKEVNA-----NFEYQASELTEESTELTAHIKFECSAEKLIFELKSR
Q5DZY2_VIBF1/18-94    AEMGFVIKEGDDVAEVSIFA-DNKVLLESEFSNYLALAKEVCA-----DVEHSIV-ITEDEKELHARLKFSCSAEKLIYELKTR
A0KF70_AERHH/20-98    AEIGTIIGEGDDVMELVIEA-AAEADARAKLAAYEALAKQVTA---EATSSSELSQG-ANGVILKARLQFTCTAEKLIFEMRAR
A4SSP9_AERS4/20-98    AEIGTIIGEGDDLMELVIEA-ATEADARAKLAAYEALAKQVTA---EAMISSELSQG-ASGVILKARLQFTCTAEKLIFELRAR
A6FGT2_9GAMM/17-93    AEIGSIIHPDDTELSFPISF-DSQLAVDEKRSELEA---YVEEQFDEA-VTINFTAQ-DEHTYL-VTLSFTCTAEKMIFEMNLR
Q1ZBI9_9GAMM/20-98    VELGSVINADDTVFVLPFKG-SSLDGVNKMAKKYIDAAKKRFE---SVIISTEETQK-EEIFHIEVTFTFECTAEKLIFEMGLS
A1STA8_PSYIN/65-96    --------------------------------------------------EQEIT-A-DDLVTLEVTFNFDCTAEKLIFEMGLS
A0KX72_SHESA/23-94    VDIGAVIDEQDTMLELSFAG-A---QAEVEASKMLERAQARFS-----QTQGNIVKN-DAGVTLK--LVFDVSVEKMIFQLENG
Q0HVE7_SHESR/23-94    VDIGAVIDEQDTVLELSFAG-A---EAEVEATKMLERAQARFS-----QTQGNIVQD-EAGVTLK--LVFDVSVEKMIFQLENG
Q0HII8_SHESM/23-94    VDIGAVIDEQDTVLELSFAG-A---EAEVEATKMLERAQARFS-----QTQGNIVQD-EAGVTLK--LVFDVSVEKMIFQLENG
```

```
A8FUY8_9GAMM/23-94     ADIGAVIDEHDTQLVVSFTGDAAQAEAEAMSAKAKERFEEIKT-----EIKSENSEV-------TLLLNFAFSAEKMIFQLENG
A3QEM6_SHELP/23-94     ADIGAVIDEFDTVLTLVREG-DK---AQSELQALAEAAKQRFE-----GVESDIKAV-EGALEL--RLTFGYSAEKMIFQLEHG
A6T9S1_KLEPN/15-96     IDVGTIIDNEDCVYRAEKVF-PNREEAESTVAAVRERAAAAAPASEPPQVDYTIVAA-GDAVKLDLSIAFSCQAEKIIFELSLR
Q31U68_SHIBS/15-96     IDIGTVIDNDNCTSKFLRFF-ATREEAESFMTKLKELAAAASSADEGASVAYKIKDL-EGQVELDAAFTFSCQAEMIIFELSLR
Q8FBC6_ECOL6/15-96     IDIGTVIDNDNCTSKFSRFF-ATREEAESFMTKLKELAAAASSADEGASVAYKIKDL-EGQVELDAAFTFSCQAEMIIFELSLR
A7ZUD6_ECOLX/15-96     IDIGTVIDNDNCTSKFSRFF-ATREEAESFMTKLKELAAAASSADEGASVAYKIKDL-EGQVELDAAFTFSCQAEMIIFELSLR
Q3YV56_SHISS/15-96     IDIGTVIDNDNCTSKFSRFF-ATREEAESFMTKLKELAAAASSADEGASVAYKIKDL-EGQVELDAAFTFSCQAEMIIFELSLR
A1AI98_ECOK1/15-96     IDIGTVIDNDNCTSKFSRFF-ATREEAESFMTKLKELAAAASSADEGASVAYKIKDL-EGQVELDAAFTFSCQAEMIIFELSLR
Q0SY67_SHIF8/15-96     IDIGTVIDNDNCTSKFSRFF-ATREEAESFMTKLKELAAAASSADEGASVAYKIKDL-EGQVELDAAFTFSCQAEMIIFELSLR
Q8X7A3_ECO57/15-96     IDIGTVIDNDNCTSKFSRFF-ATREEAESFMTKLKELAAAASSADEGASVAYKIKDL-EGQVELDAAFTFSCQAEMIIFELSLR
Q0TAE2_ECOL5/15-96     IDIGTVIDNDNCTSKFSRFF-ATREEAESFMTKLKELAAAASSADEGASVAYKIKDL-EGQVELDAAFTFSCQAEMIIFELSLR
Q1R3Z6_ECOUT/15-96     IDIGTVIDNDNCTSKFSRFF-ATREEAESFMTKLKELAAAASSADEGASVAYKIKDL-EGQVELDAAFTFSCQAEMIIFELSLR
Q32A88_SHIDS/15-96     IDIGTVIDNDNCTSKFSRFF-ATREEAESFMTKLKELAAAASSADEGASVAYKIKDL-EGLVELDAAFTFSCQAEMIIFELSLR
A2UFB3_ECOLX/15-96     IDIGTVIDNDNCTSKFSRFF-ATREEAESFMTKLKELAATSSADEGVSVAYKIKDL-EGQVELDAAFTFSCQAEMIIFELSLR
Q5I292_9ENTR/15-96     IDIGTVIDNDNCTSKFSRFF-ATREEAESFMTKLKELAATSSADEGASVAYKIRDL-EGQVELDAAFTFSCQAEMIIFELSLR
YIIS_ECOLI/15-96       IDIGTVIDNDNCTSKFSRFF-ATREEAESFMTKLKELAATSSADEGASVAYKIKDL-EGQVELDAAFTFSCQAEMIIFELSLR
A8A727_ECOLX/15-96     IDIGTVIDNDNCTSKFSRFF-ATREEAESFMTKLKELAATSSADEGASVAYKIKDL-EGQVELDAAFTFSCQAEMIIFELSLR
Q32DJ2_SHIDS/23-102    MDVGTIMDNSDCTASYSRVF-ANRAEAEQTLAALTEKARSVES--EPCKISPTFTEE-SDGVRLDVDFTFACEAEMLIFQLGLR
Q7UC47_SHIFL/23-102    MDVGTIMDNSDCTASYSRVF-ANRAEAEQTLAALTEKSRSVES--EPCKISPTFTEE-SDGVRLDIDFTFACEAEMLIFQLGLR
Q0T2E4_SHIF8/15-94     MDVGTIMDNSDCTASYSRVF-ANRAEAEQTLAALTEKSRSVES--EPCKISPTFTEE-SDGVRLDIDFTFACEAEMLIFQLGLR
Q8XCN7_ECO57/23-102    MDVGTIMDNSDCTASYSRVF-ANRAEAEQTLAALTEKARSVES--EPCKISPTFTEE-SDGVRLDIDFTFACEAEMLIFQLGLR
A8A2L2_ECOLX/15-94     MDVGTIMDNSDCTASYSRVF-ANRAEAEQTLAALTEKARSVES--EPCKISPTFTEE-SDGVRLDIDFTFACEAEMLIFQLGLR
Q31YB5_SHIBS/23-102    MDVGTIMDNSDCTASYSRVF-ANRAEAEQTLAALTEKARSVES--EPCKISPTFTEE-SDGVRLDIDFTFACEAEMLIFQLGLR
Q3YZM0_SHISS/23-102    MDVGTIMDNSDCTASYSRVF-ANRAEAEQTLAALTEKARSVES--EPCKISPTFTEE-SDGVRLDIDFTFACEAEMLIFQLGLR
A7ZPG0_ECOLX/15-94     MDVGTIMDNSDCTASYSRVF-ANRAEAEQTLAALTEKARSVES--EPCKISPTFTEE-SDGVRLDIDFTFACEAEMLIFQLGLR
A2UDM3_ECOLX/15-94     MDVGTIMDNSDCTASYSRVF-ANRAEAEQTLAALTEKARSVES--EPCKISPTFTEE-SDGVRLDIDFTFACEAEMLIFQLGLR
YFCZ_ECOLI/15-94       MDVGTIMDNSDCTASYSRVF-ANRAEAEQTLAALTEKARSVES--EPCKITPTFTEE-SDGVRLDIDFTFACEAEMLIFQLGLR
Q1R970_ECOUT/23-102    MDVGTIMDNSDCTASYSRVF-ANRAEAEQTLAALTEKARSVES--EPCKITPTFTEE-SDGVRLDIDFTFACEAEMLIFQLGLR
Q0TFA4_ECOL5/15-94     MDVGTIMDNSDCTASYSRVF-ANRAEAEQTLAALTEKARSVES--EPCKITPTFTEE-SDGVRLDIDFTFACEAEMLIFQLGLR
YFCZ_ECOL6/15-94       MDVGTIMDNSDCTASYSRVF-ANRAEAEQTLAALTEKARSVES--EPCKITPTFTEE-SDGVRLDIDFTFACEAEMLIFQLGLR
A1ADJ0_ECOK1/23-102    MDVGTIMDNSDCTASYSRVF-ANRAEAEQTLAALTEKARSVES--EPCKITPTFTEE-SDGVRLDIDFTFACEAEMLIFQLGLR
A8ADP0_CITK8/1-80      MDVGTIMDNSDCTASYSRVF-ENRAQAEETLAALTEKARGVES--EPCQITSTFTEE-AEGVRLDIDFVFACEAETLIFQLGLR
Q8XG86_SALTI/15-94     MDVGTIMDNSDCTASYSRVF-ATRAEAEETLAALTEKARSVES--EPCQITPTFTEE-SEGVRLDIDFVFACEAETLIFQLGLR
Q57LW4_SALCH/23-102    MDVGTIMDNSDCTASYSRVF-ATRAEAEETLAALTEKARSVES--EPCQITPTFTEE-SEGVRLDIDFVFACEAETLIFQLGLR
Q7CQ33_SALTY/15-94     MDVGTIMDNSDCTASYSRVF-ATRAEAEETLAALTEKARSVES--EPCQITPTFTEE-SEGVRLDIDFVFACEAETLIFQLGLR
Q5PLF9_SALPA/15-94     MDVGTIMDNSDCTASYSRVF-ATRAEAEETLAALTEKARSVES--EPCQITPTFTEE-SEGVRLDIDFVFACEAETLIFQLGLR
A6TC21_KLEPN/1-80      MDVGTIVDNTDCTASYSRVF-ANRAEAEQTLAALSEKARNVES--EPCQINPTFTDV-DGGVQLDIDFVFSCEAESLIFQLGLR
A7MH79_ENTS8/15-94     IDVGTIIDNTDCVASYSRVF-ENRADAEATLAALTAKAREVES--EPCQITPRFTEE-ADGVRLDIDFVFACQAETVIFQLGLR
A4WCW8_9ENTR/15-94     MDVGTIMDNTDCTASYSRVF-SKRADAEETLSALTKRARDVES--DPCEIKSTLTEV-EGGVKLDIDFIFACEAETLIFQLGLR
A1JK21_YERE8/24-103    IDVGTIMDNTDCTASYSCVF-DNRVEAEAMLKTLTEKARAVES--EPCLIEHKLEET-DGGVRLTVDFTFACQAETMIFQLGLR
A7FGJ9_YERPS/19-98     VDVGTVMDNTDRTASYSCVF-ASRHEAEAMLKTLTDKARAVES--DPCLIEHKLEDV-EGGVRLTIDFTFACQAETMIFQLGLR
A6BNL4_YERPE/19-98     VDVGTVMDNTDRTASYSCVF-ASRHEAEAMLKTLTDKARAVES--DPCLIEHKLEDV-EGGVRLTIDFTFACQAETMIFQLGLR
Q1C658_YERPA/19-98     VDVGTVMDNTDRTASYSCVF-ASRHEAEAMLKTLTDKARAVES--DPCLIEHKLEDV-EGGVRLTIDFTFACQAETMIFQLGLR
Q8D0U9_YERPE/21-100    VDVGTVMDNTDRTASYSCVF-ASRHEAEAMLKTLTDKARAVES--DPCLIEHKLEDV-EGGVRLTIDFTFACQAETMIFQLGLR
Q1CHK0_YERPN/19-98     VDVGTVMDNTDRTASYSCVF-ASRHEAEAMLKTLTDKARAVES--DPCLIEHKLEDV-EGGVRLTIDFTFACQAETMIFQLGLR
Q0WDE6_YERPE/19-98     VDVGTVMDNTDRTASYSCVF-ASRHEAEAMLKTLTDKARAVES--DPCLIEHKLEDV-EGGVRLTIDFTFACQAETMIFQLGLR
Q668U9_YERPS/19-98     VDVGTVMDNTDRTASYSCVF-ASRHEAEAMLKTLTDKARAVES--DPCLIEHKLEDV-EGGVRLTIDFTFACQAETMIFQLGLR
A4TM84_YERPP/19-98     VDVGTVMDNTDRTASYSCVF-ASRHEAEAMLKTLTDKARAVES--DPCLIEHKLEDV-EGGVRLTIDFTFACQAETMIFQLGLR
Q6D2L5_ERWCT/19-98     VDVGTIMDNTDCTASYSNVF-GDRSDAEATLAALTEKARAVES--EPCEIASTLEDV-DGGVKLDIDFTFSCQVETMIFQLGLR
A8GH88_9ENTR/19-98     VDVGTVMDNTDCTASYSQVF-TNQQDAEKMLAALTEKARGVES--DPCDISSSIKPV-DGGVELVADFTFACQAETFIFQLGLR
Q2NSG9_SODGM/19-98     VDVGTIIDNEDCKASFSRAF-AERQQAESMLARLTEKARAVES--DPCQIDARIDPQ-DNGFVLDADFTFCCQAETLIFQLGLR
Q0I5D5_HAES1/19-98     ADVGTIIDNSELSVDFSQVY-ENETTAQEALAYLTEKARKAET--ESCDIRSEIKEV-NGNYQLNATFTFSCQAETLIFQLSIR
A5EW75_DICNV/45-124    VEIGTIIDGKDCTVDVDYHY-DNKGLAQKALDYFTEKARAAES--EPCRIKSEIIES-AHGAQLKAQFTFSCQAEAMIFQLSTR
Q9CLX9_PASMU/29-108    VDVGTIIDGSDCTVEFEQVY-ASQAQAEEALAYLTEKAKAAES--DPCEITSDISAV-EQGALLKAQFVFSCQAEAMIFQLSTR
A4N2S2_HAEIN/16-93     VDVGTIIDGSDCSVEVHQFY-STEADANVALERLTKKARDTES--DPCEIKSEIVAV-ENGVQLNASFTFSCQAEAMI--LNWR
Consensus/80%           h-lG.lbcps-ss.phpbhh.ssc.php..b..bhpbAp.spt.....plp.ph....psthpLphpFpF.spAE.bIFpL.hR
```

| | | |
|---|---|---|
| Residue groups | Group: | sequence(consensus) |
| | Negative: | DE (-) |
| | Ser/Thr: | ST (*) |
| | Aliphatic: | ILV (l) |
| | Positive: | HKR (+) |
| | Tiny: | AGS (t) |
| | Aromatic: | FHWY (a) |
| | Charged: | DEHKR (c) |
| | Small: | ACDGNPSTV (s) |
| | Polar: | CDEHKNQRST (p) |
| | Big: | EFHIKLMQRWI (b) |
| | Hydrophobic: | ACFGHILMTVWY (h) |

**Figure S1**.

Multiple sequence alignment of the 123 proteins of Pfam domain family PF04175 with the sequences of proteins VPA0419 (SwissProt/TrEMBL ID Q87J34_VIBPA) and yiiS (SwissProt/TrEMBL ID Q83IT9_SHIFL) placed at the top. The figure has been prepared with the program CHROMA.[1]

**Additional information on the search for proteins being structurally similar to VPA0419 and yiiS.**

Proteins VPA0419 and yiiS exhibit a frequently encountered architecture (see Fig. 1): the search of the PDB[2] using either DALI[3] or SKAN[4,5] returned an extended list of structurally similar proteins. This indicates that this (rather simple) arrangement of two helices being attached to a three-stranded β-sheet represents a common motif is structure space and therefore a search for structurally similar homologues of proteins VPA0419 and yiiS (that is, proteins sharing common ancestry with VPA0419 and yiiS) also depends on functional considerations.

As discussed in the paper, analysis of the top ranked 'structural hits' lead us to hypothesize that an evolutionary link exists between proteins VPA0419 / yiiS and single domain Pterin-4a-Carbinolamine Dehydratases (PCDs).[6] Briefly, PCDs are enzymes which are involved in recycling oxidized pterin cofactors generated by aromatic amino acid hydroxylases.[7] Furthermore, PCDs are known to be bifunctional proteins in mammals, additionally acting as dimerization cofactors of the hepatocyte nuclear factor 1-alpha.[8,9] Also, it has recently been proposed that some of the PCD-like proteins found in plants and prokaryotes may in fact play a different functional role.[7]

In SCOP,[10] the PCD-like superfamily is classified together with the C-terminal domain of arginine repressor, RBP11-like subunits of RNA polymerase and GAD domain superfamilies as 'DCoH-like'. In general, these DCoH-like proteins feature the same topology as VPA0419 and yiiS albeit with an extra β-strand located at the N-terminus resulting in a four-stranded β-sheet. Moreover, many members of the PCD-like superfamily exhibit an extra N-terminal α-helix, which, however is not present in the PCD from *Thermus thermophilus* (1usm) alluded to in the paper (note, that in UniProt[11] this protein is annotated as a full length protein).

Although proteins VPA0419 and yiiS exhibit structural similarity with most of the DCoH-like proteins, comparison of surface and catalytic site residues suggest a more direct evolutionary link only for PCDs. The structure of PCD from *Rattus Norvegicus* (PDB ID 1dcp) crystallized in complex with the product analogue

7,8-dihydrobiopterin[8] provides details about ligand binding in the active site of the enzyme. Among the residues that participate in ligand binding, five highly conserved ones were identified by site-directed mutagenesis to be vital to preserve enzymatic activity (that is, His 62, His 63, Pro 64, His 80 and Asp 89; Fig. S2a). These residues are located in a cavity formed by (i) the loop connecting α-helix I and β-strand B, (ii) the C-terminal segment of β-strand C, and (iii) the N-terminal segment of helix II (note: the regular secondary structure elements in the PCD are named here as the structurally aligned elements of proteins VPA0419 and yiiS).

While the catalytic residues of the PCDs are themselves not conserved in proteins VPA0419 and yiiS (Fig. S2a), it is remarkable that several residues which can be structurally aligned with the PCD's active site are well-conserved in protein family PFAM 04175 (Fig. S1, S2). In fact, those include the strictly conserved residues Glu 89 and Phe 84 (residue numbers are as for protein yiiS), along with Ala 63 (Cys in many other proteins of PFAM 04175), Ser 57 (Asp or Cys in other proteins of PFAM 04175), Cys 86 (Val in other proteins of PFAM 04175) (Figs. S1, S2c). Furthermore, the only other surface region of protein VPA0419 and yiiS exhibiting significant residue conservation is located on α-helix II, and most of the conserved residues are hydrophobic (Ala 88, Ile 91, Ile 92, Leu 95 in protein yiiS; Fig. S2b). As concluded in the paper, this 'suggests that in both VPA0419 and yiiS (and therefore all members of PF04175) this region (i.e. the one including Glu 89) is involved in binding of a ligand, thereby possibly constituting a catalytic site of a yet uncharacterized enzyme specific to *gamma proteobacteria*.'

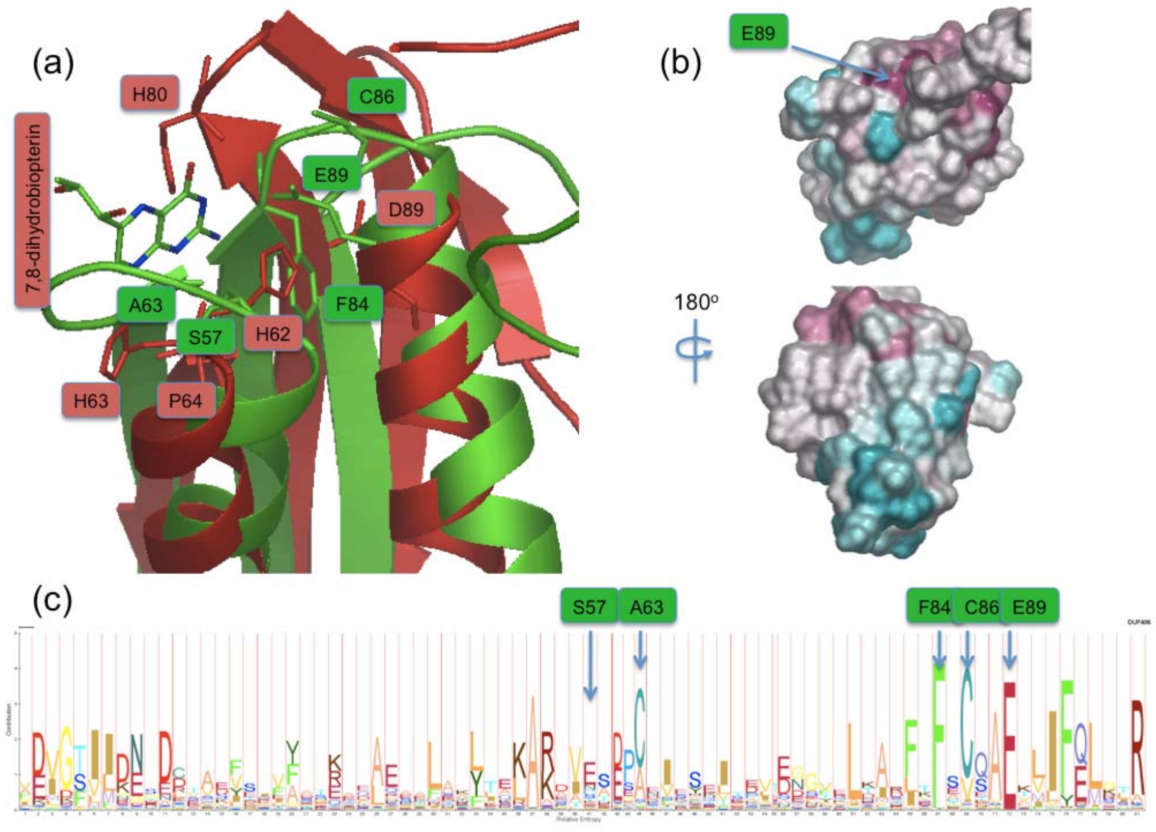


**Figure S2**

(a) Ribbon-drawing presentation of the structural superposition of PCD from *Rattus Norvegicus* (PDB ID 1dcp) (red)[8] and protein yiiS (PDB ID 2k3i) (green). Conserved residues in the family of PCD sequence homologues which are involved in 4a-carbinolamine binding are indicated and shown as a cylinder representation (red). Residues of protein yiiS which are conserved in protein family PFAM 04175 are indicated and shown as a cylinder representation (green). The product analogue 7,8-dihydrobiopterin (co-crystallized with the PCD) is also indicated and shown as a cylinder representation (CPK colors).

(b) Conservation of surface residue of protein yiiS in protein family PFAM 04175 which can be structurally aligned with 4a-carbinolamine binding site of the PCD. The top figure is in the same orientation as (a), the bottom figure is rotated by 180 degrees about the axis shown in the figure. In the top figure, the location of residue Glu 89 is indicated by an arrow. The figure was generated using the program ConSurf[12] with the input of the multiple sequence alignment for PF04175 (Fig. S1). Color code ranges from purple (conserved) to cyan (not conserved).

(c) Pfam HMM logo[13] displaying the residue conservation in protein family PF04175. Conserved residues shown in (a) are highlighted.

## REFERENCES


1. Goodstadt L, Ponting CP. CHROMA: consensus-based colouring of multiple alignments for publication. Bioinformatics 2001;17:845-846.
2. Berman HM, Westbrook J, Feng Z, Gilliland G, Bhat TN, Weissig H, Shindyalov IN, Bourne PE. The Protein Data Bank. Nucleic Acids Res 2000;28:235-242.
3. Holm L, Sander C. Dali: a network tool for protein structure comparison. Trends Biochem Sci 1995;20:478-480.
4. Petrey D, Honig B. GRASP2: visualization, surface properties, and electrostatics of macromolecular structures and sequences. Methods Enzymol 2003;374:492-509.
5. Yang AS, Honig B. An integrated approach to the analysis and modeling of protein sequences and structures. I. Protein structural alignment and a quantitative measure for protein structural distance. J Mol Biol 2000;301:665-678.
6. Zhao G, Xia T, Song J, Jensen RA. Pseudomonas aeruginosa possesses homologues of mammalian phenylalanine hydroxylase and 4 alpha-carbinolamine dehydratase/DCoH as part of a three-component gene cluster. Proc Natl Acad Sci U S A 1994;91:1366-1370.
7. Naponelli V, Noiriel A, Ziemak MJ, Beverley SM, Lye LF, Plume AM, Botella JR, Loizeau K, Ravanel S, Rebeille F, de Crecy-Lagard V, Hanson AD. Phylogenomic and functional analysis of pterin-4a-carbinolamine dehydratase family (COG2154) proteins in plants and microorganisms. Plant Physiol 2008;146:1515-1527.
8. Cronk JD, Endrizzi JA, Alber T. High-resolution structures of the bifunctional enzyme and transcriptional coactivator DCoH and its complex with a product analogue. Protein Sci 1996;5:1963-1972.
9. Suck D, Ficner R. Structure and function of PCD/DCoH, an enzyme with regulatory properties. FEBS Lett 1996;389:35-39.
10. Murzin AG, Brenner SE, Hubbard T, Chothia C. SCOP: a structural classification of proteins database for the investigation of sequences and structures. J Mol Biol 1995;247:536-540.
11. Apweiler R, Bairoch A, Wu CH, Barker WC, Boeckmann B, Ferro S, Gasteiger E, Huang H, Lopez R, Magrane M, Martin MJ, Natale DA, O'Donovan C, Redaschi N, Yeh LS. UniProt: the Universal Protein knowledgebase. Nucleic Acids Res 2004;32:D115-119.
12. Landau M, Mayrose I, Rosenberg Y, Glaser F, Martz E, Pupko T, Ben-Tal N. ConSurf 2005: the projection of evolutionary conservation scores of residues on protein structures. Nucleic Acids Res 2005;33:W299-302.
13. Schuster-Bockler B, Schultz J, Rahmann S. HMM Logos for visualization of protein families. BMC Bioinformatics 2004;5:7.
